# Probing the habitual and compulsive-like basis of (dys)functional checking in the Observing Response Task, a rodent analogue relevant to obsessive-compulsive disorder

**DOI:** 10.1007/s00213-026-07094-9

**Published:** 2026-05-22

**Authors:** Luise Pickenhan, Amy L. Milton

**Affiliations:** Department of Psychology, University of Cambridge Downing Site, CB2 3EB Cambridge, United Kingdom

**Keywords:** Checking, Observing Response Task, Obsessive-Compulsive Disorder, Rat, Contingency degradation, Punishment

## Abstract

**Rationale:**

Excessive and maladaptive checking is a prominent symptom of obsessive-compulsive disorder (OCD). This dysfunctional checking can be modelled using the Observing Response Task (ORT), which distinguishes between functional and dysfunctional checking shown by the same individuals, in rodents and humans. We have previously observed that rats classified as sign-trackers on a pavlovian autoshaping procedure show elevated levels of dysfunctional checking.

**Objectives:**

We sought to determine whether dysfunctional checking was habitual and compulsive.

**Methods:**

119 Lister Hooded rats (M = 95, F = 24) were classified using pavlovian autoshaping and concurrently underwent ORT training. Briefly, rats learned to respond on one of two levers to receive reinforcement, with the correct lever changing unpredictably throughout the session. Pressing a third, ‘observing’ lever illuminated a light cue over the currently correct lever. These functional Observing Lever Presses (OLPs) can be distinguished from dysfunctional Extra Observing Lever Presses (eOLPs), which have no programmed consequences. Whether dysfunctional checking was habitual was examined by degrading the contingency between checking and light cue presentation, and whether it was compulsive was determined by punishing 50% of checking responses.

**Results:**

Sign-trackers showed higher levels of dysfunctional checking than goal-trackers and intermediates, including after contingency degradation. Punished checking eradicated checking behaviour in most rats, but a minority of sign-trackers persisted despite punishment via shock.

**Conclusions:**

These data support the hypothesis that sign-trackers exhibit more cue-driven checking behaviour than goal-trackers. Observations from punished checking refer to the prevalence of severe OCD in the human population concerning persistence of pathological checking despite aversive consequences.

**Supplementary information:**

The online version contains supplementary material available at 10.1007/s00213-026-07094-9.

## Introduction

Obsessive-Compulsive Disorder (OCD) is a debilitating neuropsychiatric condition with an average lifetime prevalence of 3.5%, affecting 5.4% of women and 1.7% of men (Angst et al. 2004; Fawcett et al. 2020). OCD is characterised by two cardinal symptom clusters; obsessions and compulsions. Obsessions denote unwanted, intrusive, and recurring thoughts, mental images, or impulses that patients struggle to suppress, whereas compulsions describe repetitive and stereotyped behaviours that the individual feels compelled to perform (Van Oppen et al. 1995). Compulsions in OCD can be conceptualised as an imbalance between goal-directed behaviour and habitual learning. Habits can be adaptive, in that they facilitate behaviour to occur automatically without impinging on cognitive load that would otherwise be recruited by more conscious goal-directed decision-making (Haith and Krakauer 2018; Wood and Rünger 2016; Friston et al. 2014). However, habits become maladaptive when compulsive; when individuals develop pathological urges to carry out certain behaviours without functional reasons for doing so (Graybiel and Rauch 2000; Gillan et al. 2011). This has detrimental consequences for affected individuals’ everyday functioning and mental well-being and is emblematic of the behavioural aberrations seen in OCD (Gillan et al. 2014).

To elucidate the behavioural mechanisms and fundamental learning processes underlying compulsive phenomena in OCD, such as excessive checking, animal models of dysfunctional checking have been developed. After the first rodent model of quinpirole-induced checking was put forward by Szechtman et al. (1998), Eagle et al. (2014) introduced the Observing Response Task (ORT; described in Pickenhan and Milton 2024). The ORT is an appetitive instrumental procedure whose design allows for the distinction between functional and dysfunctional versions of the same checking behaviour, and aims to clarify the shift from goal-directed to habitual to ostensibly compulsive-like checking behaviour in rodents. It has been shown repeatedly, in male rats, that ‘sign-trackers’, classified separately on a pavlovian autoshaping procedure, are more prone to excessive levels of dysfunctional checking (Eagle et al. 2020; Vousden et al. 2020; McKenzie et al. 2025). This is consistent with the increased control over behaviour by reward-based cues – often characterised as ‘incentive salience’ – shown in sign-trackers (Robinson and Flagel 2009; Olney et al. 2018; Srey et al. 2015). Importantly, sign-tracking has been correlated with increased reliance on habitual learning systems (Lesaint et al. 2015) and linked to higher levels of compulsive behaviour (Albertella et al. 2019).

To determine whether checking on the ORT is habitual, this study tested the impact of degrading the contingency between the checking response and its informative outcome in two different types of probe test – one in which checking produced *no* cue outcome (i.e. under extinction conditions) and one in which checking produced an *uninformative* outcome (with light presentation no longer above the currently correct lever). These manipulations therefore decoupled checking from both its informational value (in both conditions) and any value that the cue might have accrued as a conditioned reinforcer (under extinction conditions).

This study also sought to determine whether checking is compulsive, at least in some individuals. While it has previously been shown that checking on the ORT can be modulated by perceived threat (via the presentation of shock-associated cues) and actual threat (by punishing incorrect responses) (Vousden et al. 2020), it remains unknown whether checking *itself* is punishment-resistant (and therefore compulsive). To test this, we developed a novel punished version of the Observing Response Task (pORT), in which checking was probabilistically reinforced with either the informative light cue or an electric foot shock. We hypothesised that, if sign-trackers are more likely to show an increased predisposition towards compulsivity (Flagel et al. 2009; Robinson et al. 2013) and similar to the punishment-resistance observed in animals compulsively seeking drugs of abuse (Pelloux et al. 2007; Torres et al. 2017), that sign-tracking rats would show greater resistance to punishment on the punished version of the ORT. This would draw parallels to an extensive body of research on compulsivity in addiction, expand its implications for animal models of OCD, and provide the first study elucidating the impact of punishment on both functional and dysfunctional checking behaviour in rodents. Thus, this study examined whether checking on the ORT was habitual and compulsive in male and, for the first time, female rats.

## Methods

### Subjects

Subjects were 119 (24 female) behaviourally naïve Lister Hooded rats (Charles River, UK), aged at least 8 weeks at the start of behavioural testing. Animals were housed in groups of three rats per cage under a reversed light-dark cycle (lights off at 07:00 and on at 19:00, with 30-minute ‘dusk’ and ‘dawn’ periods prior to these times) with a cardboard tube as enrichment. Rats were allowed to acclimatise to the animal facility for at least seven days prior to the start of any experimental procedures. Testing was carried out daily, typically between 7:00 h and 13:00 h. Prior to the start of behavioural procedures, animals were food-restricted such that they were maintained at 90–95% of their age-matched free-feeding body weight, being fed standard laboratory chow (SDS) upon completion of each day’s testing, in addition to any food reward earned during behavioural sessions. This research was regulated under the Animals (Scientific Procedures) Act 1986 Amendment Regulations 2012 following ethical review and approval by the University of Cambridge Animal Welfare and Ethical Review Body (AWERB) and was conducted under Project Licences PA9FBFA9F and PP9536688.

### Materials & apparatus

All behavioural procedures took place in 18 operant conditioning chambers (Med Associates Inc., Vermont, USA) equipped with three retractable levers, two of which flanked a food receptacle for the delivery of 45 mg sucrose pellets (TestDiet, OpCoBe, UK). A stimulus light was placed above each of these two levers as well as centrally above the food magazine. A third lever, the Observing Response lever, was located on the opposite wall of the operant conditioning chambers, above which there was a white house light that remained illuminated throughout the session. Each chamber was enclosed within a sound-attenuating box. Chamber operation and online data collection were controlled and monitored via WhiskerControl Server software (Cardinal and Aitken 2010) and the Observing Response Task programme, written originally by A.C. Mar and modified for the punished checking task by J.C. Beanland, and the LeverAutoshaping programme, written by R.N. Cardinal.

### Behavioural procedures

Rats were trained on the Observing Response Task (ORT) as described previously (Eagle et al. 2014; Vousden et al. 2020). Briefly, rats were trained on the ORT and underwent separate autoshaping training sessions to enable classification into goal-tracking, intermediate and sign-tracking phenotypes. Animals were subsequently tested in the ORT under conditions of uncertainty (uORT), where it was more difficult for animals to discriminate between the currently active and inactive levers. To test whether checking behaviour was habitual, rats underwent two different tests of contingency degradation with rebaselining sessions of uORT in between. To test whether checking behaviour was resistant to punishment, rats underwent several sessions of ORT with punished checking (pORT) in which checking was probabilistically reinforced with an electric footshock of varying magnitude across sessions.

#### Observing Response Task

Rats were trained on the ORT as previously described (Eagle et al. 2014; Vousden et al. 2020). Rats were trained to distinguish active from inactive levers as both front-panel levers were presented, with one being active whilst the other one was inactive. The identity of the active and inactive levers changed throughout the session on a variable time (VT) 30s schedule (range: 15–45 s) during initial training. The identity of the currently active lever was signalled by illumination of a cue light above the lever. The observing lever remained retracted during these initial sessions. Responses on the active lever were reinforced initially on a fixed ratio (FR) 1 schedule by presentation of a sucrose pellet in the food magazine (45 mg TestDiet sucrose pellets, OpCoBe, UK), before advancing through progressively leaner FR and variable ratio (VR) schedules based on performance. Ultimately, all rats progressed to responding on a VR15 (range: 10–20) schedule. These behavioural training sessions were initially conducted twice daily with a break of approximately 25 minutes between sessions, followed by one of those two sessions being substituted by a pavlovian autoshaping session as the first one for each testing day.

Upon consistent discrimination of the active versus the inactive levers, as well as completion of 17 sessions of autoshaping, rats underwent training on first the full, and second the ‘uncertainty’ versions of the ORT. For both versions of the task, the observing lever was presented, with responses on the observing lever illuminating the cue light above the currently active lever. This illumination period of the cue light above the active lever lasted for 30 s during the first four sessions of full ORT training, and 15 s during all subsequent sessions. Active lever presses were reinforced on a VR15 schedule (range: 10–20), including changes to the identity of active and incorrect levers every 90 s (FT90s). Following ten sessions of the full version of the ORT, rats underwent 15 sessions of the uncertain version of the ORT (uORT). This was the same as the full ORT, except that the active lever was reinforced on a variable interval (VI10-20s) rather than VR10-20, and the switch time between levers changed from VI15-45s to VI20-120s seconds. Responses on the task were automatically recorded by a computer-based WhiskerControl Server (Cardinal and Aitken 2010). Seven dependent variables were measured during the ORT session: (i) observing lever presses (OLPs), which were functional observing responses leading to illumination of the light above the currently active lever; (ii) extra observing lever presses (eOLPs), which were responses on the observing lever performed when the cue light was already illuminated; (iii) active lever presses (rate per minute); (iv) inactive lever presses (rate per minute); (v) discrimination with the cue light on; (vi) discrimination with the cue light off, and; (vii) reward pellets earned.

#### Pavlovian autoshaping

Previous reports have found that sign-tracking rats show elevated levels of dysfunctional checking (Vousden et al. 2020; Eagle et al. 2020), and so rats were screened using a pavlovian autoshaping procedure to classify sign-trackers (STs), goal-trackers (GTs), and intermediates (INTs). During each of the 17 autoshaping sessions, the lever that would subsequently be used as the observing lever was presented 30 times, in the absence of the two levers on the front of the chamber. Ten seconds after presentation of the lever it retracted, which coincided with delivery of a single sucrose pellet (45 mg TestDiet pellets, OpCoBe, UK) into the food magazine on the other side of the chamber. Data were automatically recorded by the computer, and rats were classified as sign-trackers, goal-trackers, or intermediates by an experimenter who remained blind to their ORT performance. Classification occurred as described in Eagle et al. (2014) and Vousden et al. (2020), where the number of lever approaches and the number of nose pokes made during the conditioned stimulus (CS) were used to calculate a ratio of conditioned stimulus : magazine approaches. This ratio was averaged across the final two sessions of autoshaping, and the distribution ratios plotted. Clear subpopulations were observed in the distributions, with group differences observed in CS approaches and magazine approaches (**Supplementary Figs. **1** and **2). The data were cross-checked with previous classifications using this method to ensure consistency across cohorts.

#### Contingency degradation

Upon completion of uORT, rats underwent contingency degradation by exposure to two probe tests, presented in a counterbalanced order across animals. In the *Extinction Probe*, the test ran in the same manner as the uORT except that the cue light remained off throughout the entire session, thereby removing any information provided regarding the location of the currently active lever. In the *Uninformative Probe*, pressing of the observing lever illuminated the cue light located centrally between the two levers, above the food magazine, again removing any information provided regarding the location of the currently active lever, but still providing an *uninformative* outcome of observing lever pressing. Both approaches for implementing contingency degradation thus represent devaluation procedures, whereby the observing lever was devalued by either removing the cue light indicating the identity of the active lever for subsequent pellet delivery as a secondary reinforcer entirely (extinction), or removing the informational content of the cue light upon observing lever pressing by means of illuminating the middle cue light instead of the active lever (uninformative cue presentation).

#### Punished checking

Following completion of contingency degradation and subsequent rebaselining on the uORT, 95 rats (24 females) that had undergone contingency degradation probe tests were tested on the punished ORT (pORT). This version of the task ran in the same manner as the uORT, except that presses on the observing lever were probabilistically reinforced with the cue light on 50% of trials, and with delivery of a scrambled electric footshock (0.5s, 0.1–0.5 mA, varying across sessions) on 50% of trials. Each press on the observing lever, whether classified as an OLP or eOLP, had the same probability of eliciting shock. Following initial runs of animals displaying no behavioural changes with 0.1 mA and 0.2 mA foot shocks, subsequent runs were tested with only 0.3–0.5 mA shocks. The data for 0.1 mA and 0.2 mA shock levels can be found in the **Supplementary Materials**. Rats were considered to be ‘punishment resistant’ if they showed dysfunctional checking during the 0.5 mA shock session.

### Statistical analysis

Data are presented as means ± standard error of the mean (SEM). Autoshaping data (**Supplementary Materials**; CS approaches and nose pokes) were analysed using a mixed model analysis of variance (ANOVA), with Session as a within-subjects factor and Phenotype (GT, ST or INT) as a between-subjects factor. ORT contingency degradation probe test data were analysed with mixed model ANOVAs, with Session as a within-subjects factor, and Phenotype (GT, ST or INT) and Sex as between-subjects factors. Order (Extinction or Uninformative probe being presented first) was included in initial analyses but subsequently removed as a factor due to no significant effects found on primary or secondary measures of task performance (see **Supplementary Materials**). Punished ORT data were analysed with mixed model ANOVAs, with shock magnitude (ShockMag) as a within-subjects factor, and Phenotype and Sex as between-subjects factors. Rates of active and inactive lever pressing served as measures of generalised task performance, to determine whether alterations in checking might be attributable to other, more general behavioural effects such as reduced locomotion or motivation to seek reward. Rates of active and inactive lever pressing were calculated by dividing the total number of active or inactive lever presses by the length of the session (21 min) and rates of pressing were compared with mixed model ANOVAs with Lever and either Session or ShockMag as within-subjects factors, and Phenotype and Sex as between-subjects factors. Discrimination was calculated by dividing the number of active lever presses during the relevant period (cue light on or off) by the total number of active and inactive lever presses during that same period, and multiplying by 100 to generate a percentage. For the extinction probe session, the ‘on’ period was counted as the 15 s following an observing lever press (though no cue light was presented). For punished checking, the ‘on’ period was counted as the 15 s following an observing lever press. Where no lever presses were made during the relevant period, discrimination values were manually set to zero. Discrimination data were analysed with mixed model ANOVAs with Cue (on or off) and either Session or ShockMag as within-subjects factors, and Phenotype and Sex as between-subjects factors. Where Mauchly’s test of sphericity was violated, the Greenhouse-Geisser correction was applied when ε < 0.75 and the Huynh-Feldt correction when ε > 0.75. A Chi-squared test was used to test the numbers of animals of each phenotype that were ‘punishment resistant’ and ‘punishment sensitive’, defined by dysfunctional checking during the 0.5 mA shock session. Any significant effects were further examined using pairwise comparisons, using the Šidák correction for multiple comparisons.

## Results

### Behavioural phenotyping

Carrying out behavioural classification of 119 Lister hooded rats on a pavlovian autoshaping task into either sign-trackers (ST), goal-trackers (GT), or intermediates (INT) yielded the following sample sizes (Table 1) for each phenotype and sex (**Supplementary Figs. **1** and **2). These aligned with distributions observed in previous studies using the ORT in male rodents (Eagle et al. 2020; Vousden et al. 2020).

**Table 1:** Phenotypic composition in total numbers and relative percentages for male and female Lister Hooded rats, respectively, and for both cohorts combined, following pavlovian autoshaping

Phenotype	Males	Females	Total N (M + F)
Goal-trackers	47 [49.5%]	4 [16.7%]	51 [42.8%]
Intermediates	26 [27.3%]	9 [37.5%]	35 [29.4%]
Sign-trackers	22 [23.1%]	11 [45.8%]	33 [27.7%]
Total N	95	24	119

### Sign-trackers and goal-trackers performed differently on tests of contingency degradation

**Fig. 1:**
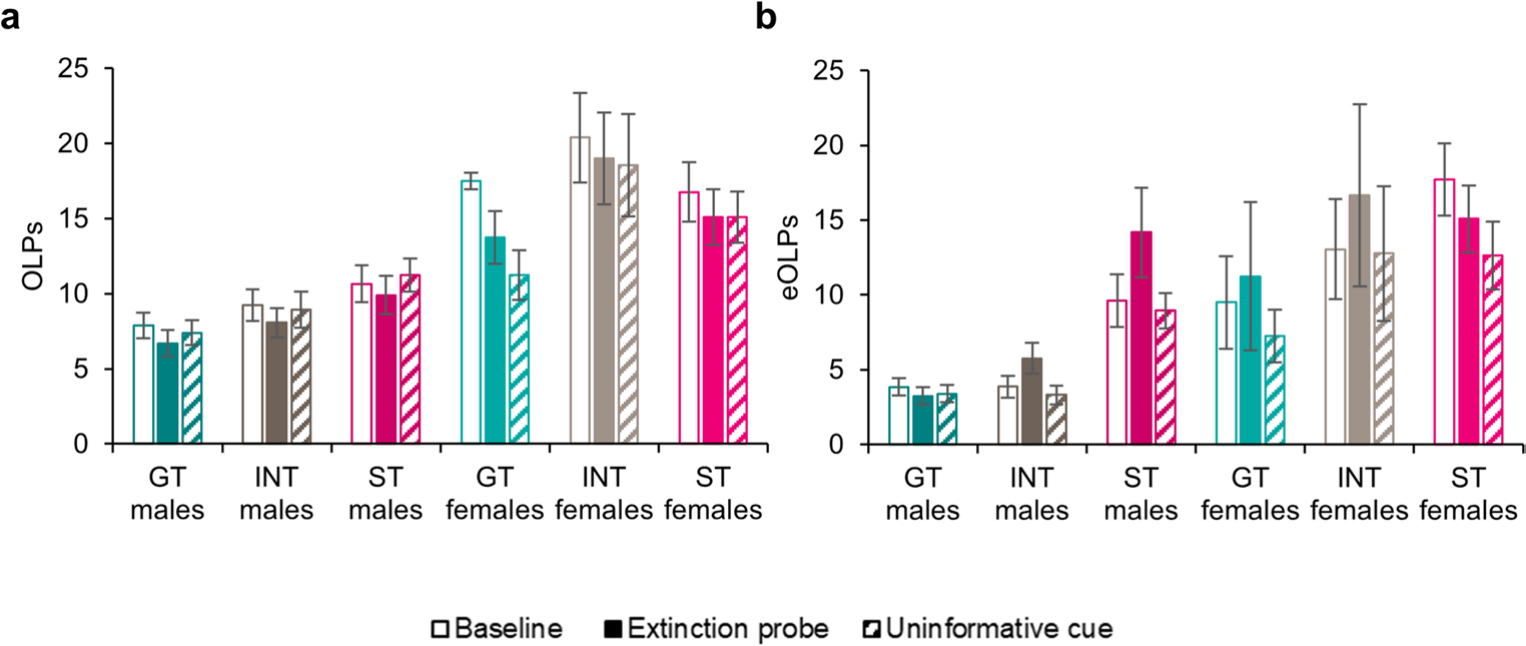
(**a**) Observing lever presses (OLPs) and (**b**) extra observing lever presses (eOLPs) made by male and female goal-trackers (GTs), intermediates (INTs) and sign-trackers (STs) during baseline ORT sessions and during the extinction and uninformative probe tests. Data are shown as means ± SEM. Group sizes: male GTs, *n* = 47; male INTs, *n* = 26; male STs, *n* = 22; female GTs, *n* = 4; female INTs, *n* = 9; female STs, *n* = 11

Sign-trackers performed differently to goal-trackers and intermediates in terms of functional (Fig. 1a) and dysfunctional (Fig. 1b) checking on two measures of contingency degradation.

#### Functional checking

For functional observing lever presses (OLPs; Fig. 1a), a mixed factorial ANOVA revealed that females showed greater levels of responding than males [Sex: *F*_(1,113)_ = 22.9, *p* < .001, η_p_^2^ = 0.17] though the overall pattern of responding was similar between the sexes [Session x Sex: *F*_(1.38,157)_ = 1.98, *p* = .16; Session x Sex x Phenotype: *F*_(2.78,157)_ = 1.02, *p* = .38]. Responding differed in between the averaged baseline session and the two different tests of contingency degradation [Session: *F*_(1.38,157)_ = 5.37, *p* = .013, η_p_^2^ = 0.045], with Šidák-corrected pairwise comparisons showing that OLPs were higher in the averaged baseline sessions than the two tests of contingency degradation (both *p*’s = 0.003) but that responding did not differ between the two tests of contingency degradation (*p* > .99). However, this overall effect of responding obscured differences in responding when the animals were divided by phenotype [Session x Phenotype: *F*_(2.78,157)_ = 3.08, *p* = .033, η_p_^2^ = 0.052]. While goal-trackers showed reduced responding relative to baseline for both the extinction probe test (*p* = .005) and the uninformative probe test (*p* = .048), neither intermediate nor sign-tracking rats showed reduced responding on either test (all *p*’s > 0.25). Thus, although there were no overall differences in OLPs made during the baseline and contingency degradation tests by sign-trackers, intermediates or goal-trackers [Phenotype: *F*_(2,113)_ = 2.43, *p* = .093], goal-trackers tended to reduce their functional checking during contingency degradation, while intermediates and sign-trackers did not.

Thus, similar to our previous observations with perceived threat (Vousden et al. 2020), goal-trackers reduced their functional checking in a flexible manner, while sign-trackers did not.

#### Dysfunctional checking

For dysfunctional extra observing lever presses (eOLPs; Fig. 1b) a similar analysis showed higher levels of lever pressing in females [Sex: *F*_(1,113)_ = 19.3, *p* < .001; η_p_^2^ = 0.15]. Again, the overall pattern of responding was similar between the sexes [Session x Sex: *F* < 1; Phenotype x Sex: *F*_(2,113)_ = 1.55, *p* = .22]. Consistent with our previous observations, sign-trackers showed higher levels of dysfunctional eOLPs [Phenotype: *F*_(2,113)_ = 6.41, *p* = .002, η_p_^2^ = 0.10] with sign-trackers showing higher levels of dysfunctional checking than goal-trackers (*p* = .003) but not intermediates (*p* = .062). There were also differences in the levels of dysfunctional checking shown by males and females of different phenotypes during the contingency degradation sessions [Session x Phenotype x Sex: *F*_(3.57,202)_ = 2.55, *p* = .047, η_p_^2^ = 0.043]. While there were no differences in the number of dysfunctional presses made by male or female goal-trackers or intermediate animals during either contingency degradation probe test compared to baseline (all *p*’s > 0.21), male sign-trackers made more eOLPs during the extinction probe test than at baseline (*p* = .002) while female sign-trackers did not (*p* = .39). Furthermore, female sign-trackers reduced their eOLPs relative to baseline during the uninformative probe test (*p* = .002) while male sign-trackers did not (*p* = .89).

Thus, as for OLPs, female rats showed greater levels of dysfunctional checking overall, and different effects of contingency degradation manipulations on eOLPs. While sign-trackers checked more than intermediates and goal-trackers of the same sex, male sign-trackers *increased* their dysfunctional checking during the extinction probe test but not during the uninformative cue probe test. By contrast, female sign-trackers did not alter responding during the extinction probe test, but instead *reduced* responding during the uninformative cue probe test. Given the unbalanced numbers of male versus female animals tested for this study, analyses were repeated with the male-only dataset, yielding results congruent with previously reported analyses except for intermediate rats reducing their functional checking during the extinction probe test compared to performance at baseline (*p* = .022), similar to goal-tracking rats rather than sign-trackers, with the latter described above.

### Generalised measures of task performance during contingency degradation

#### Active and inactive lever pressing

**Fig. 2:**
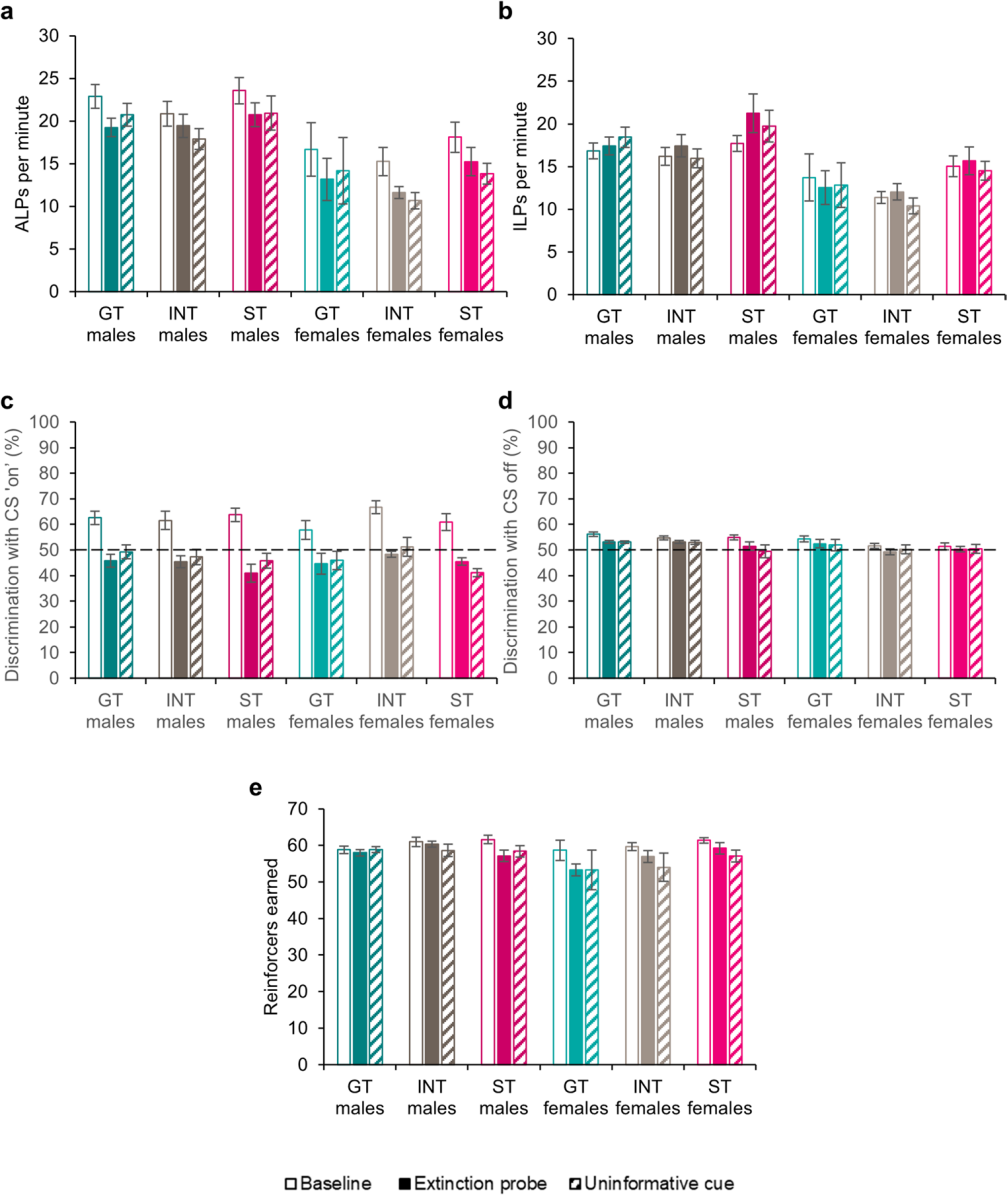
(**a**) Active lever pressing (ALPs) per minute and (**b**) inactive lever pressing (ILPs) per minute for male and female goal-trackers (GTs), intermediates (INTs) and sign-trackers (STs). (**c**) Discrimination between the active and inactive levers with the light cue ‘on’ (though this was off during the extinction probe test) and (**d**) discrimination between the active and inactive levers with the light cue off during the baseline, extinction probe and uninformative cue sessions. Chance responding is 50% and is denoted by a dotted line. (**e**) Reinforcers earned during the baseline, extinction probe and uninformative cue probe tests. Data are shown as means ± SEM. Group sizes: male GTs, *n* = 47; male INTs, *n* = 26; male STs, *n* = 22; female GTs, *n* = 4; female INTs, *n* = 9; female STs, *n* = 11

All rats pressed the active (Fig. 2a) more than the inactive (Fig. 2b) lever [Lever: *F*_(1,113)_ = 30.5, *p* < .001, η_p_^2^ = 0.21], though overall responding was lower in the uninformative probe test compared to the baseline and extinction probe sessions [Session: *F*_(2,226)_ = 4.29, *p* = .015, η_p_^2^ = 0.04; Lever x Session: *F*_(1.95,220)_ = 16.7, *p* < .001, η_p_^2^ = 0.13]. While the rates of inactive lever pressing did not differ between the baseline and contingency degradation sessions (all *p*’s > 0.40), rates of active lever pressing were higher during the baseline session compared to the two contingency degradation probe tests (*p*’s < 0.001), but the rates of active lever pressing did not differ between the extinction probe and uninformative cue probe tests (*p* = .98). In contrast to their checking, where they responded *more* than males, females showed lower rates of lever pressing [Sex: *F*_(1,113)_ = 12.5, *p* < .001, η_p_^2^ = 0.99] particularly on the active lever [Lever x Sex: *F*_(1,113)_ = 5.11, *p* = .026, η_p_^2^ = 0.04; pairwise comparisons showed reduced pressing compared to males on both the active (*p* < .001) and the inactive (*p* = .002) levers].

There were no differences in rates of active and inactive lever pressing by goal-trackers, intermediates or sign-trackers [Phenotype: *F*_(2,113)_ = 1.66, *p* = .19; Lever x Phenotype: *F* < 1; Session x Phenotype: *F* < 1; Lever x Session x Phenotype: *F* < 1], and within each sex, there were no differences between goal-trackers, intermediates and sign-trackers [Phenotype x Sex: *F* < 1; Lever x Phenotype x Sex: *F* < 1; Session x Phenotype x Sex: *F* < 1; Lever x Phenotype x Session x Sex: *F* < 1].

#### Cue discrimination with the light cue ‘on’ and off during contingency degradation

Discrimination between the active and inactive levers was calculated for each session, comparing discrimination while the cue light was on (or the equivalent time period following an OLP for the extinction session) and off. There was a marked effect of session, with discrimination being better when the cue was on during the baseline sessions than the probe test sessions [Session: F_(2,226)_ = 30.1, *p* < .001, η_p_^2^ = 0.21; Cue x Session: *F*_(2,226)_ = 18.7, *p* < .001, η_p_^2^ = 0.14]. This was further supported by Šidák-corrected pairwise comparisons, where discrimination (with cue on or off) during the baseline session differed from the probe tests (both *p*’s < 0.001), which did not differ from each other (*p* = .95).

Discrimination did not differ between males and females [Sex: *F* < 1; Session x Sex: *F* < 1; Cue x Session x Sex: *F* < 1] or between goal-trackers, intermediates and sign-trackers [Phenotype: *F*_(2,113)_ = 1.36, *p* = .26; Session x Phenotype: *F* < 1; Cue x Session x Phenotype: *F* < 1] and there were no interactions [Phenotype x Sex: *F* < 1; Session x Phenotype x Sex: *F* < 1; Cue x Session x Phenotype x Sex: *F* < 1]. Thus, all animals were using the cue to guide responding during the baseline sessions (when it was both available and informative) but, as expected, discrimination declined during the probe sessions when the cue was either not present (extinction probe) or uninformative.

#### Reinforcers earned

All rats earned more reinforcers (Fig. 2e) in the baseline session, when the cue was present and informative, compared to the extinction probe and the uninformative cue probe sessions [Session: *F*_(1.93,218)_ = 13.5, *p* < .001, η_p_^2^ = 0.11; pairwise comparisons showed that rewards earned in the baseline session were higher than the probe tests (both *p*’s < 0.001), where the rewards earned did not differ (*p* = .73)]. There were no differences in the numbers of reinforcers earned by males and females [Sex: *F*_(1,113)_ = 2.33, *p* = .13; Session x Sex: *F*_(1.93,218)_ = 2.67, *p* = .073] and no differences between goal-trackers, intermediates and sign-trackers [Phenotype: *F* < 1; Session x Phenotype: *F* < 1]. There were also no differences between male and female goal-trackers, intermediates or sign-trackers [Phenotype x Sex: *F* < 1; Session x Phenotype x Sex: *F*_(3.86,218)_ = 1.07, *p* = .38]. Thus, despite their lower rates of active lever pressing, females earned as many reinforcers as males, and the numbers of reinforcers earned were similar across the population.

### Assessing the compulsive-like nature of (dys)functional checking behaviour with punishment

#### Functional checking

**Fig. 3:**
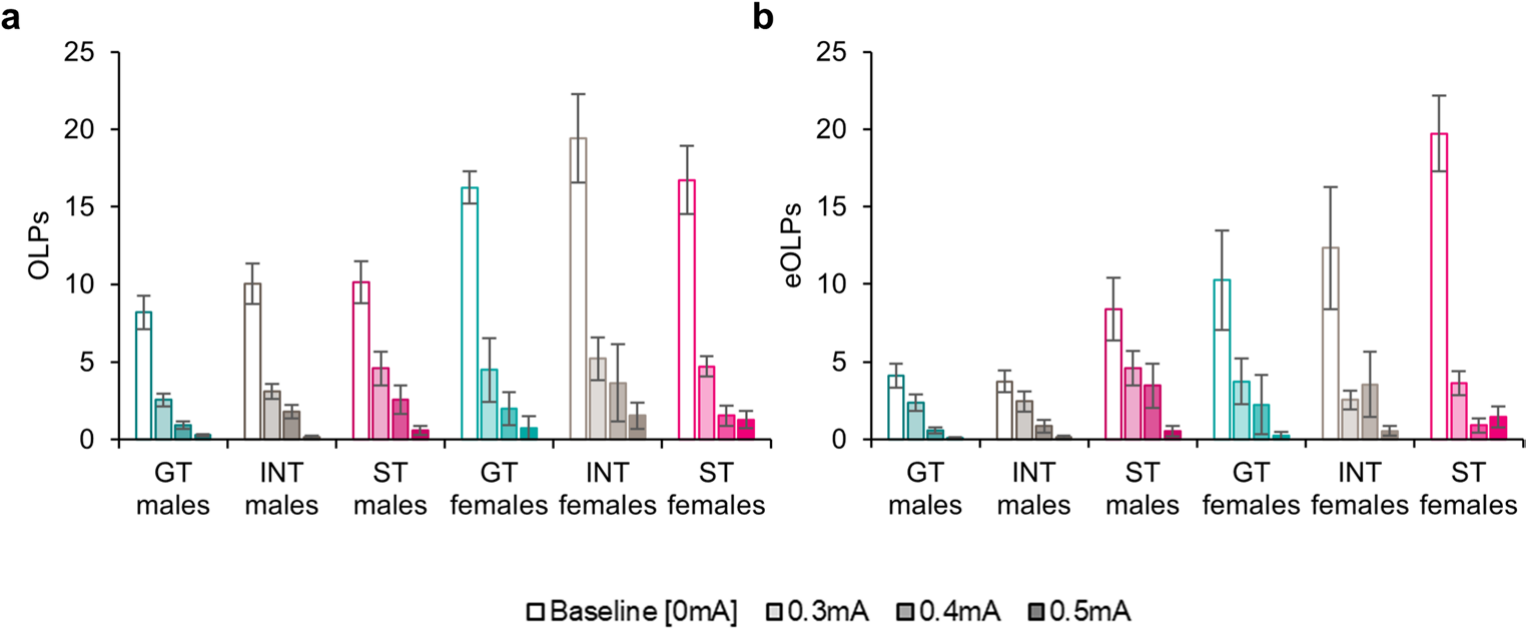
(**a**) Observing lever presses (OLPs) and (**b**) extra observing lever presses (eOLPs) made by male and female goal-trackers (GTs), intermediates (INTs) and sign--trackers (STs) during baseline ORT sessions and sessions in which checking was punished with varying magnitudes of shock (mA). Data are shown as means ± SEM. Group sizes: male GTs, *n* = 36; male INTs, *n* = 20; male STs, *n* = 15; female GTs, *n* = 4; female INTs, *n* = 9; female STs, *n* = 11

As was seen for the contingency degradation manipulations, female rats showed higher levels of functional checking [Fig. 3a; Sex: *F*_(1,89)_ = 18.1, *p* < .001, η_p_^2^ = 0.17]. All rats reduced their functional checking as the shock magnitude was increased [ShockMag: *F*_(1.73,154)_ = 171, *p* < .001, η_p_^2^ = 0.66] with differences in the effect of shock magnitude on OLPs in males and females [ShockMag x Sex: *F*_(1.73,154)_ = 16.3, *p* < .001, η_p_^2^ = 0.15]. While males reduced their OLPs as the shock magnitude increased (comparing the shock magnitudes with Šidák-corrected pairwise comparisons, all *p*’s < 0.004), for females the numbers of OLPs made when punished with the 0.4 mA and 0.5 mA shock magnitudes did not differ (*p* = .16).

There were no differences in the number of OLPs elicited under punishment for goal-trackers, intermediates or sign-trackers [Phenotype: *F*_(2,89)_ = 1.06, *p* = .35; ShockMag x Phenotype, *F* < 1] and no differences between the phenotypes within each sex [Phenotype x Sex: *F*_(2,89)_ = 1.15, *p* = .32; ShockMag x Phenotype x Sex: *F* < 1].

#### Dysfunctional checking

As was the case for OLPs, females made greater numbers of eOLPs (Fig. 3b) on the punished ORT than males [Sex: *F*_(1,89)_ = 17.9, *p* < .001, η_p_^2^ = 0.17]. Although all rats reduced their dysfunctional checking as the shock magnitude was increased [ShockMag: *F*_(1.88,167)_ = 71.6, *p* < .001, η_p_^2^ = 0.45], the effect of shock magnitude on dysfunctional checking varied by both sex [ShockMag x Sex: *F*_(1.88,167)_ = 18.3, *p* < .001, η_p_^2^ = 0.17] and between goal-trackers, intermediates and sign-trackers [ShockMag x Phenotype: *F*_(3.76,167)_ = 4.45, *p* = .002, η_p_^2^ = 0.09]. While females made greater numbers of eOLPs at baseline (*p* < .001), once the shock contingencies were introduced their responding became equivalent to that of the males at the 0.3 mA (*p* = .83) and 0.4 mA (*p* = .47) shock magnitudes, though with a trend towards higher eOLPs under the 0.5 mA shock condition (*p* = .056).

There were also differences in responding across goal-trackers, intermediates and sign-trackers of both sexes [Phenotype: *F*_(2,89)_ = 7.11, *p* = .001, η_p_^2^ = 0.14; Phenotype x Sex: *F* < 1; ShockMag x Phenotype x Sex: *F*_(3.76,167)_ = 1.96, *p* = .11]. As we have reported previously, STs showed higher levels of dysfunctional checking (both *p*’s < 0.006) than intermediates or goal-trackers, who did not differ from each other (*p* = .97). Comparing the performance of goal-trackers, intermediates and sign-trackers under different conditions of punished checking, sign-trackers showed greater levels of dysfunctional checking both under baseline conditions (all *p*’s < 0.005) and in the 0.5 mA condition, with greater dysfunctional checking than goal-trackers (*p* = .041) but not intermediates (*p* = .74). However, under the 0.3 mA and 0.4 mA conditions, there were no differences between the phenotypes (all *p*’s > 0.21). Thus, sign-trackers showed higher levels of dysfunctional checking when there were no adverse consequences (i.e. no shocks) and were able to reduce their checking under moderate levels of shock (0.3 mA and 0.4 mA). However, at levels of shock that suppressed dysfunctional checking in intermediates and goal-trackers (0.5 mA), sign-trackers continued to show low levels of dysfunctional checking.

Classifying rats as punishment-resistant if they showed dysfunctional checking during the 0.5 mA shock session revealed differences between the three phenotypes. While 8/26 sign-trackers showed dysfunctional checking during this session (30.8% of the sign-tracking population), 5/29 (17.2%) of intermediates and 3/40 (7.5%) of goal-trackers showed dysfunctional checking during this session [χ^2^_(2,*n*=95)_ = 6.10, *p* = .047].

### Generalised measures of task performance during punished checking

#### Active and inactive lever pressing

**Fig. 4:**
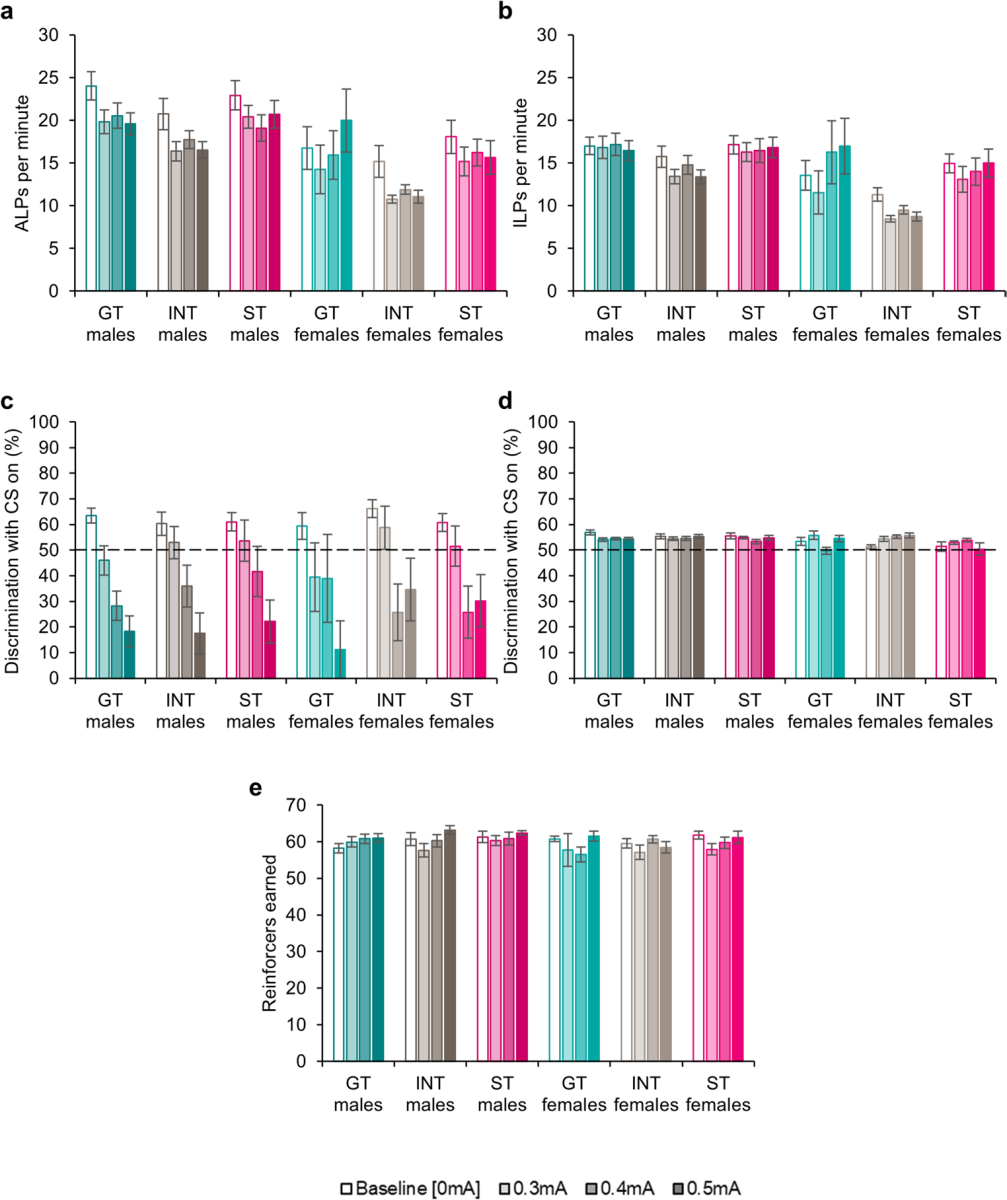
(**a**) Active lever pressing (ALPs) per minute and (**b**) inactive lever pressing (ILPs) per minute for male and female goal-trackers (GTs), intermediates (INTs) and sign-trackers (STs). (**c**) Discrimination between the active and inactive levers with the light cue on and (**d**) discrimination between the active and inactive levers with the light cue off during sessions in which checking was punished with varying magnitudes of shock (mA). Chance responding is 50% and is denoted by a dotted line. (**e**) Reinforcers earned during the baseline, extinction probe and uninformative cue probe tests. Data are shown as means ± SEM. Group sizes: male GTs, *n* = 36; male INTs, *n* = 20; male STs, *n* = 15; female GTs, *n* = 4; female INTs, *n* = 9; female STs, *n* = 11

All rats showed higher rates of pressing on the active (Fig. 4a) lever than the inactive (Fig. 4b) lever [Lever: *F*_(1,89)_ = 107, *p* < .001, η_p_^2^ = 0.55] and across the population, responding decreased with increasing shock magnitude [ShockMag: *F*_(2.37,211)_ = 6.86, *p* < .001, η_p_^2^ = 0.07] with greater reductions on the rate of active lever pressing [Lever x ShockMag: *F*_(2.00,178)_ = 52.6, *p* < .001, η_p_^2^ = 0.09]. As was the case for the tests of contingency degradation, female rats pressed the active and inactive levers less than males [Sex: *F*_(1,89)_ = 7.89, *p* = .006, η_p_^2^ = 0.08; Sex x Lever: *F*_(1,89)_ = 7.31, *p* = .008, η_p_^2^ = 0.08]. Pairwise comparisons revealed that females showed lower rates of both active (*p* = .003) and inactive (*p* = .017) pressing than males, though both sexes pressed the active lever more than the inactive (all *p*’s < 0.001). This did not appear attributable to differences in shock sensitivity, as males and females were similarly affected by shock [ShockMag x Sex: *F*_(2.37,211)_ = 1.64, *p* = .19; Lever x ShockMag x Sex: *F* < 1].

The omnibus ANOVA indicated that rates of active and inactive lever pressing did vary by phenotype [Phenotype: *F*_(2,89)_ = 3.33, *p* = .04, η_p_^2^ = 0.07] though differences between phenotypes did not survive correction for multiple comparisons (all *p*’s > 0.076). There were also no significant interactions [Phenotype x Sex: *F* < 1; Lever x Phenotype: *F* < 1; Lever x Phenotype x Sex: *F* < 1; ShockMag x Phenotype: *F*_(2.37,211)_ = 1.40, *p* = .25; ShockMag x Phenotype x Sex: *F*_(4.75,211)_ = 1.09, *p* = .37; Lever x ShockMag x Phenotype: *F* < 1; Lever x ShockMag x Phenotype x Sex: *F* < 1]. Thus, overall, all animals responded more on the active over the inactive lever, reducing their rates of responding with increasing shock magnitude.

#### Cue discrimination with the light cue on and off during punished checking

Discrimination between the active and inactive levers was better in the presence of the light cue (Fig. 4c) for the baseline sessions, but rapidly reduced as rats stopped making observing responses at higher shock magnitudes [Cue: *F*_(1,89)_ = 30.5, *p* < .001, η_p_^2^ = 0.26; Cue x ShockMag: *F*_(2.99,266)_ = 21.6, *p* < .001, η_p_^2^ = 0.20]. While discrimination with the light off (Fig. 4d) remained approximately at chance for all sessions, with no differences between sessions, regardless of shock magnitude (Šidák-corrected pairwise comparisons, all *p*’s > 0.42), discrimination with the light on reduced with increasing shock magnitude, with a trend towards decreased discrimination during the 0.3 mA-punished session (*p* = .057) and reduced discrimination with the light on for the 0.4 mA and 0.5 mA sessions (all *p*’s < 0.019). There were, however, no differences in discrimination between phenotypes [Phenotype: *F* < 1; Cue x Phenotype: *F* < 1; ShockMag x Phenotype: *F* < 1] and no differences between males and females [Sex: *F* < 1; Cue x Sex: *F* < 1; ShockMag x Sex: *F* < 1]. There were also no interactions [Phenotype x Sex: *F* < 1; Cue x Phenotype x Sex: *F* < 1; ShockMag x Phenotype x Sex: *F* < 1; Cue x ShockMag x Phenotype x Sex: *F* < 1].

#### Reinforcers earned

The numbers of reinforcers earned (Fig. 4e) varied across the shock sessions [ShockMag: *F*_(2.96,263)_ = 3.63, *p* = .014, η_p_^2^ = 0.04]. However, this was not due to lower numbers of reinforcers earned during the shock sessions, as these did not differ from baseline (all *p*’s > 0.25) but instead, counterintuitively, was due to *increased* numbers of reinforcers earned during the 0.5 mA session compared to the 0.3 mA session (*p* = .01). There were no differences in the numbers of reinforcers earned by males and females [Sex: *F* < 1; ShockMag x Sex: *F* < 1] and no differences between goal-trackers, intermediates and sign-trackers [Phenotype: *F* < 1; ShockMag x Phenotype: *F* < 1] and no interactions [Phenotype x Sex: *F* < 1; ShockMag x Phenotype x Sex: *F*_(5.91,263)_ = 1.12, *p* = .35].

## Discussion

This study was the first to test both male and female rats on the Observing Response Task (ORT), and to examine whether checking on this task is habitual (via contingency degradation) and/or compulsive (via punishment of checking). As has been reported previously (Vousden et al. 2020; Eagle et al. 2020), we found that animals classified as sign-trackers following pavlovian autoshaping showed elevated dysfunctional checking, measured via extra observing lever presses (eOLPs), compared to intermediate or goal-tracking rats. Furthermore, we observed higher levels of checking in female rats than male rats, whilst also finding that female sign-trackers showed elevated dysfunctional checking compared to intermediate and goal-tracking animals of the same sex, as we have previously reported for males. However, we acknowledge that the number of female rats included in this study is lower than the number of males, and thus the reported sex differences may be underpowered and should be regarded as preliminary findings.

The habitual nature of checking was tested by implementing two different modifications of the contingency between checking and information on the ORT. In an ‘extinction’ probe test, pressing the observing lever led to no consequence (i.e. the light cue indicating the currently active lever was not presented) while in an ‘uninformative cue’ probe test, responses on the observing lever led to illumination of a central light between the two levers. The order of presentation of these two probe tests was counterbalanced between animals, and differences in responding on the probe tests were found for goal-trackers, intermediates and sign-trackers, and between the two sexes.

For male rats, the removal of the cue light in the ‘extinction’ probe test led to reduced functional checking in goal-trackers, but not for intermediates or sign-trackers. Furthermore, while goal-trackers and intermediate animals maintained low levels of dysfunctional checking (eOLPs) during the extinction probe test, sign-trackers *increased* their dysfunctional checking. By comparison, presentation of the cue light in an uninformative position did not alter either the functional or dysfunctional checking of male rats relative to their own baselines. As removal of the valued outcome reduced functional checking in goal-trackers, this likely indicates that for these animals, responding for the light cue is supported by a goal-directed association. However, the lack of effect of removing the cue light in intermediates and sign-trackers suggests that their checking behaviour may be habitual. Furthermore, the finding that presentation of the cue light in an uninformative position left behaviour relatively intact in all animals allows us to speculate that the male goal-trackers and intermediates are not checking in order to gain information, but rather for presentation of the cue light itself.

Although the performance of the female rats was broadly comparable to that of the males, some important differences were found. In contrast to the males, omission of the cue light in the ‘extinction’ probe tests produced relatively little effect on goal-trackers, intermediates or sign-trackers (suggesting that they may all be responding habitually), but presentation of the cue in an uninformative position led to a reduction in functional checking in goal-trackers but not sign-trackers. However, presentation of the uninformative cue led to an unexpected reduction in *dysfunctional* checking in sign-trackers. Thus, although both male and female goal-trackers showed evidence of sensitivity to contingency degradation (and hence inferred goal-directedness of behaviour) and male and female sign-trackers showed checking consistent with habitual behaviour, the specifics of these behaviours differed. While males appeared more sensitive to omission of the cue light, females appeared more sensitive to the loss of information encoded by the cue light’s location.

Upon completion of the contingency degradation stage of this study, the compulsive-like nature of functional and dysfunctional checking behaviour was examined via punishment of the observing lever on 50% of responses. The introduction of the punishment contingency generally reduced checking behaviour, consistent with previous reports of conditioned suppression under threat conditions (Poppen 1970; Limpens et al. 2014; Chu et al. 2024). However, there were differences observed between the phenotypes, with sign-trackers showing higher levels of dysfunctional checking under baseline conditions (i.e. when there were no adverse consequences). They suppressed their checking to levels equivalent to goal-trackers and intermediates for the intermediate shock magnitudes of 0.3 mA and 0.4 mA, but during the 0.5 mA punishment session, when goal-trackers and intermediates further suppressed their checking, sign-trackers continued.

Although the decrease in both functional and dysfunctional checking as the magnitude of punishment via foot shock intensified was observed in all phenotypic groups and both sexes, these data presented a clear pattern. Namely, goal-trackers showed the greatest decline in OLPs and eOLPs throughout all shock magnitude conditions to baseline, followed by intermediate rats. Sign-trackers represented the phenotypic group with the highest number of animals continuing to perform OLPs and eOLPs, although significantly reducing their functional and dysfunctional checking upon shock administration. Therefore, it may be argued that, given the decrease in all phenotypes’ – though particularly sign-trackers’ - dysfunctional checking behaviour relative to baseline as the shock magnitude increased, this behavioural adaptation to adverse stimuli ostensibly undermines the compulsive nature of maladaptive checking shown by rats on the ORT. Whilst we acknowledge this, we note two further points.

The first is that living with clinically severe OCD, patients may encounter situations in which they are able to ‘control’, or at least mitigate, the extent to which to which their overt behavioural compulsions manifest, analogous to ‘exposure with response prevention therapy’ in real life. Thus, depending on the individual severity of their condition and overall circumstances, people living with OCD can simultaneously be negatively affected in their daily functioning while still exerting a certain degree of control over their compulsions when the consequences of performing these are perceived as sufficiently grave. The second caveat we note is that a small proportion of rats, regardless of sex or phenotypic classification, resumed checking despite the prospect of shock administration, marked by both functional and dysfunctional checking. This small proportion (functional checkers *N* = 27/95, 28.4%; dysfunctional checkers *N* = 16/95, 16.8%) numerically exceeds the proportion of the population diagnosed with OCD, which does not undermine the ORT as a valid model with relevance to OCD, but instead alludes to the spectrum of severity in terms of OCD-related behaviours, such as maladaptive checking. Moreover, the exceptional animals whose checking persisted during the shock condition were largely represented by the female sign-tracker group, hence tentatively alluding to human data whereby, amongst the general population, women tend to show higher frequency and greater severity of OCD than men (Fawcett et al. 2020; Van Zandt et al. 2025), albeit this being subject to different sub-types of OCD beyond the realm of dysfunctional checking behaviour. Furthermore, as this study included fewer females than males, the analyses of sex differences are potentially underpowered and therefore should be interpreted with caution.

Future investigations into the behavioural mechanisms underlying functional and dysfunctional checking may benefit from utilising the ORT in conjunction with pharmacological approaches by modulating the neurochemical systems thought to subserve habitual and compulsive-like behaviour, thereby granting valuable implications for pharmacotherapies relevant to human cases of OCD.

This study tested the central hypothesis that checking would be habitual and compulsive in a subset of animals (sign-trackers) trained and tested on the ORT. Furthermore, it extended previous reports (Vousden et al. 2020; Eagle et al. 2020) by testing, for the first time, female animals on the ORT. Our predictions of increased checking by sign-trackers, based on previous reports, were supported (Eagle et al. 2020). The analysis of both sexes revealed an interesting dissociation in the reliance of males and females on the conditioned reinforcing (Dinsmoor 1983) and informational (Berlyne 1960) properties of the cue light, with dysfunctional checking in males being more affected by the omission of the cue light rather than its presentation in an uninformative location, while checking in females was unaffected by cue omission, but decreased when the cue was presented in an uninformative location. The increase in dysfunctional checking (which does not produce the cue light on the uORT) in male sign-trackers may reflect an ‘extinction burst’ in responding to acquire the cue, as under extinction conditions functional and dysfunctional checking become indistinguishable (to the animal). The decrease in dysfunctional checking in female sign-trackers when the cue was presented in an uninformative location may indicate that their checking was still under goal-directed control, with degradation of the informational value of the cue reducing their motivation to work for it. Moreover, the female sign-tracking group that was found to reduce responding on the uninformative cue probe test subsequently decreased checking under shock exposure, thus corroborating their sensitivity to punishment due to reliance on goal-directed behaviour.

This study was also the first to examine the impact of punishment on checking behaviour, finding for the majority of animals that punishment of responding on the observing lever led to reductions in checking. However, some animals continued to check despite shock, and satisfied our definition of being ‘punishment-resistant’ (i.e. continuing to check even in the 0.5 mA shock session).

A minority of sign-trackers (30.8% of the sign-tracking population) showed checking that resumed in the presence of punishment via shock, suggesting in these animals that checking may satisfy the definition of ‘compulsion’ that is typically operationalised in the addiction literature (Pelloux et al. 2007; Torres et al. 2017). However, an alternative account could be that rats, which ostensibly persisted in checking on the punished version of the ORT, did so as a means of testing the shock contingency for the current session, based on their memory of shock from prior punished sessions. This would account for the relatively low number of presses performed by ‘persistent’ checkers, though this is difficult to infer with certainty due to low baseline checking. However, even with these caveats, the data are consistent with the view that sign-trackers are more likely to rely upon habitual associations (Lesaint et al. 2015) and indicate that a further vulnerability factor may make some sign-trackers susceptible to transitioning to compulsive habits. Determining the basis of this additional vulnerability will be a key question for future research.

## Supplementary material

Below is the link to the electronic supplementary material.

Supplementary File 1 (PDF 119 KB)

Supplementary File 2 (PDF 432 KB)

## Data Availability

Data are available at 10.17863/CAM.119389.
