# Supplementary figures and images for: Probing the habitual and compulsive-like basis of (dys)functional checking in the Observing Response Task, a rodent analogue relevant to obsessive-compulsive disorder
Source: Psychopharmacology (Berl). 2026 May 22;243(5):1323–35. doi: 10.1007/s00213-026-07094-9 (PMC13242449; doi:10.1007/s00213-026-07094-9)

Supplementary Figure 1

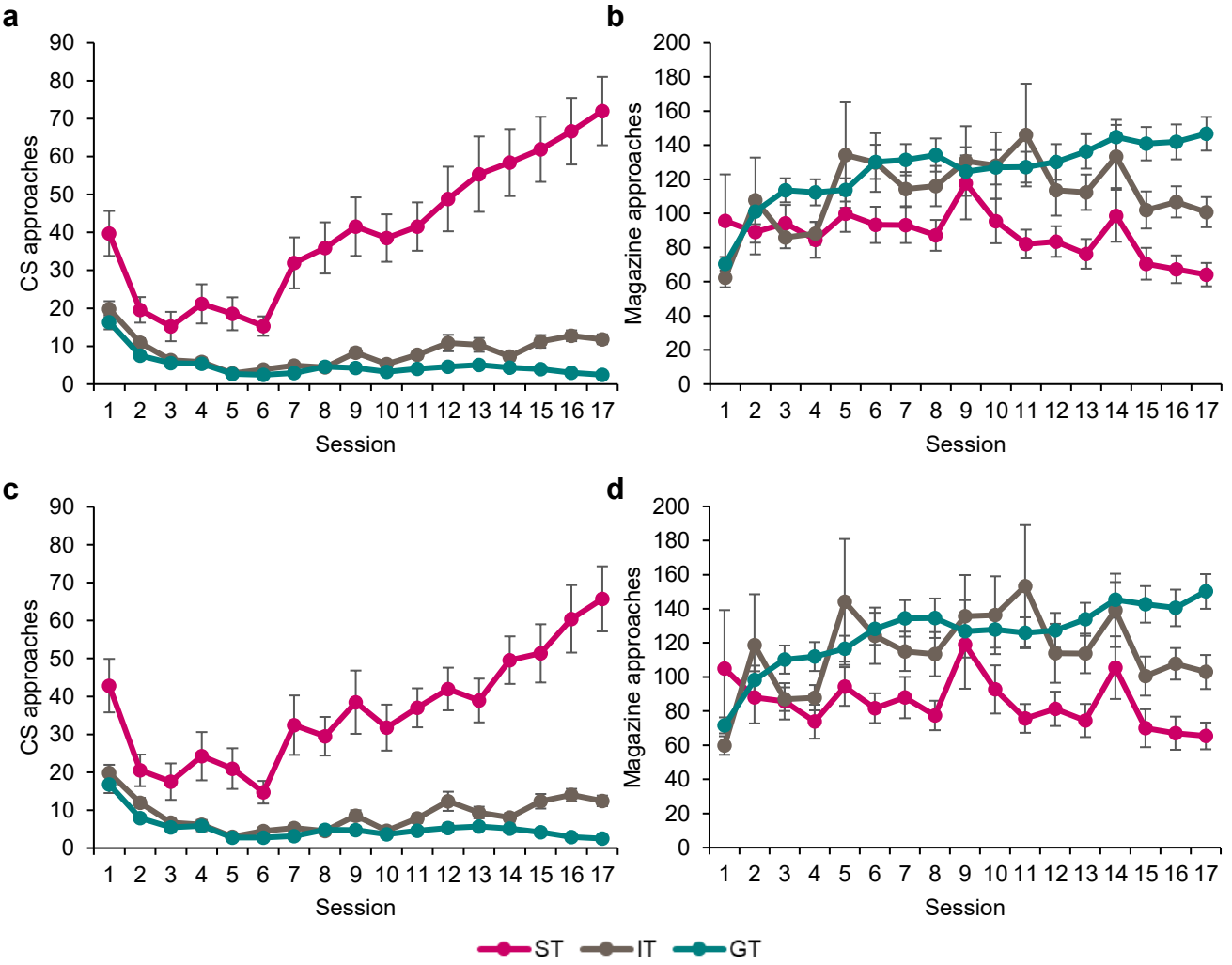

Supplementary Figure 2

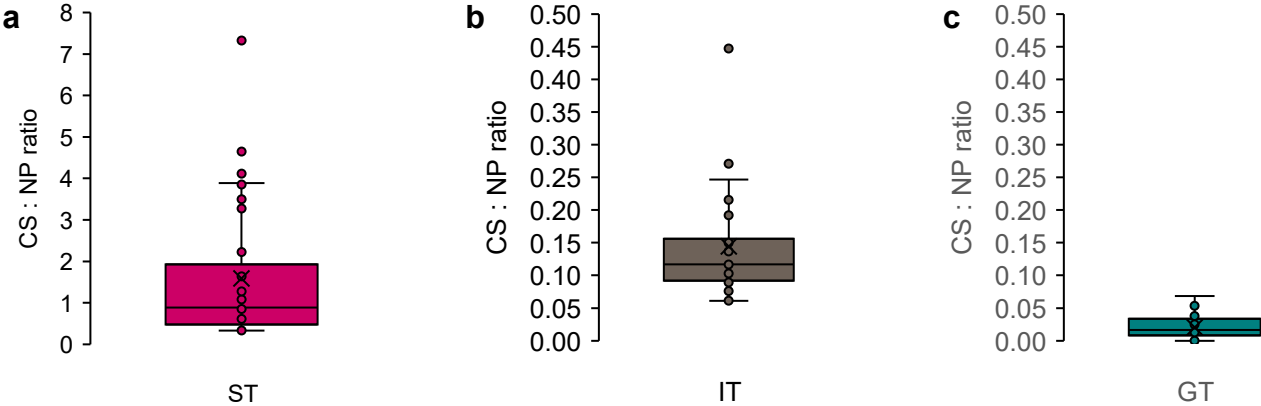

Supplementary Figure 3

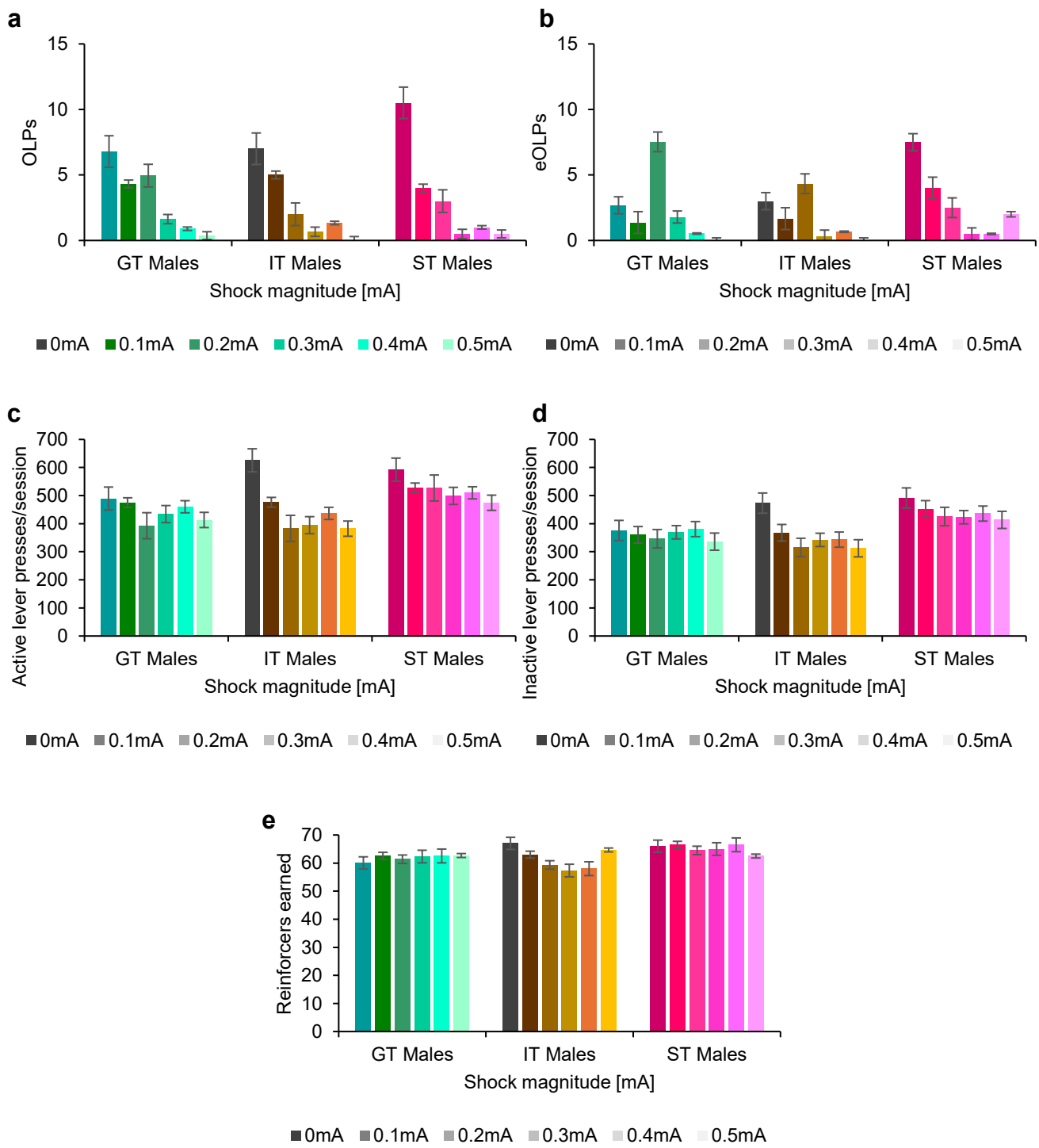

Supplement: Supplementary file 1 — (PDF 119 KB) [file 213_2026_7094_MOESM1_ESM.pdf]
