# Supplementary material for: Probing the habitual and compulsive-like basis of (dys)functional checking in the Observing Response Task, a rodent analogue relevant to obsessive-compulsive disorder
Source: Psychopharmacology (Berl). 2026 May 22;243(5):1323–35. doi: 10.1007/s00213-026-07094-9 (PMC13242449; doi:10.1007/s00213-026-07094-9)
Supplement: Supplementary file 2 — (PDF 432 KB) [file 213_2026_7094_MOESM2_ESM.pdf]

Supplementary Figure 1. Sign-trackers, intermediates and goal-trackers showed differences in CS approach (a, c) and magazine approach (b, d) during autoshaping training. All rats **(a, b)** subsequently underwent contingency degradation but as only a subset **(c, d)** subsequently underwent punished checking, data were also analysed for this subset separately. Examining autoshaping performance for all rats, **(a)** sign-trackers (ST) showed higher levels of CS approach than intermediate (IT) or goal-trackers (GT) [Phenotype: $F_{(2,116)} = 61.0$, $p < .001$, $\eta_p^2 = 0.51$]. Šidák-corrected pairwise comparisons revealed that STs made more CS approaches than either ITs or GTs (all p's < .001) but ITs and GTs showed similar levels of CS approach ($p = .54$). This difference in CS approach increased across sessions [Session: $F_{(4.37,506)} = 21.9$, $p < .001$, $\eta_p^2 = 0.16$; Session x Phenotype: $F_{(8.73,506)} = 15.1$, $p < .001$, $\eta_p^2 = 0.21$]. **(b)** GTs showed higher levels of magazine approach during autoshaping [Phenotype: $F_{(2,116)} = 3.93$, $p = .022$, $\eta_p^2 = 0.06$], with Šidák-corrected pairwise comparisons showing that GTs approached the magazine more than STs ($p = .018$) but ITs differed in their magazine approach from neither GTs ($p = .72$) or STs ($p = .24$). **(c)** As only a subset of animals progressed to punished checking, these analyses were rerun only for this subset of rats. As for the full cohort, STs in this subset showed greater CS approach than ITs and GTs [Phenotype: $F_{(2,92)} = 52.3$, $p < .001$, $\eta_p^2 = 0.53$] with Šidák-corrected pairwise comparisons revealing that STs approached the CS more than ITs and GTs (both p's < .001) but ITs and GTs showing similar levels of CS approach ($p = .54$). **(d)** Similarly, for the rats that subsequently underwent punished checking GTs showed greater magazine approach [Phenotype: $F_{(2,92)} = 3.59$, $p = .031$, $\eta_p^2 = 0.07$], with GTs approaching the magazine more than STs ($p = .029$) but not ITs ($p = .88$), which also did not differ in their approach to STs ($p = .19$).

Supplementary Figure 2. Classification of rats as STs, ITs and GTs. Rats were classified on the ratio of CS approach to magazine approach on the last two days of autoshaping training. Distributions of these averages are shown for **(a)** STs, **(b)** ITs and **(c)** GTs. Note that the ST data are presented on a different y-axis scale to allow visualisation of IT and GT data. Circles represent individual data points, the cross represents the median, and the boxes represent the interquartile ranges.

Supplementary Figure 3. Primary and secondary measures of ORT performance of a pilot sample at baseline and while undergoing punished ORT sessions with shocks administered at 0.1, 0.2, 0.3, 0.4, and 0.5mA for each phenotype. (a) Observing lever presses, (b) Extra Observing lever presses, (c) Active lever presses per session, (d) Inactive lever presses per session, and (e) number of reinforcers earned per session.
